# Participatory development of MIDY (Mobile Intervention for Drinking in Young people)

**DOI:** 10.1186/s12889-016-2876-5

**Published:** 2016-02-24

**Authors:** Cassandra J. C. Wright, Paul M. Dietze, Belinda Crockett, Megan S. C. Lim

**Affiliations:** Burnet Institute, Melbourne, Victoria Australia; Monash University, Melbourne, Victoria Australia

**Keywords:** Alcohol, Young people, Ecological Momentary Assessment, Brief intervention, Participatory design, Australia

## Abstract

**Background:**

There are few effective strategies that respond to the widespread practice of risky single-occasion drinking in young people. Brief interventions, which involve screening of alcohol consumption and personalised feedback, have shown some efficacy in reducing alcohol consumption, but are typically delivered in clinical settings. Mobile phones can be used to reach large populations instantaneously, both for data collection and intervention, but this has not been studied in combination during risky drinking events

**Methods:**

Our study investigated the feasibility and acceptability of a mobile-phone delivered Ecological Momentary Assessment (EMA) and brief intervention for young people during drinking events. Our participatory design involved development workshops, intervention testing and evaluation with 40 young people in Melbourne, Australia. The final intervention included text message prompts to fill in mobile-based questionnaires, which measured drinks consumed, spending, location and mood, with additional questions in the initial and final questionnaire relating to plans, priorities, and adverse events. Participants received a tailored feedback SMS related to their drinking after each hourly questionnaire. The intervention was tested on a single drinking occasion. Prompts were sent between 6 pm and 2 am during a drinking event, with one follow up at 12 pm the following day.

**Results:**

Participants reported being comfortable with hourly mobile data collection and intervention during social occasions, and found the level of intrusion acceptable; we achieved an 89 % response rate on the single occasion of testing. Participants were proactive in suggesting additional questions that would assist in the tailoring of feedback content, despite the added time burden. While we did not test the effectiveness of the intervention, participants reported value in the tracking and feedback process, with many stating that they would normally not be aware of how much alcohol they consumed in a night.

**Conclusions:**

Findings suggest that the intervention was considered acceptable, feasible and novel to our participants; it now requires comprehensive testing and evaluation.

## Background

In Australia, alcohol consumption is a significant public health issue; Risky Single Occasion Drinking (RSOD) (also known as binge drinking) is widespread and particularly concerning. More than one in seven deaths and one in five hospitalisations among young people are attributed to alcohol consumption, largely related to RSOD rather than long-term heavy consumption [1]. RSOD is associated with a plethora of harms including physical and sexual violence, suicide, risky sexual behaviour, as well as both short- and long-term brain impairment and cognitive deficits [1–4]. RSOD is common in Australia and persists beyond adolescence, with more than 66 % of 18- to 24-year-olds and 64 % of 25–29-year-old Australians reporting such drinking within the past year [5].

Thus far, researchers have identified few strategies that effectively reduce harmful drinking [6]. Education and information provision are historically popular for their visibility, reach and low cost, but strategies such as mass-media campaigns, health warnings and school-based programs have limited effect [6–8].

Clinical interventions, including brief screening and tailored feedback delivered as ‘brief interventions’, have strong and growing evidence of efficacy for reducing drinking [9, 10]. Brief interventions are based on ‘Motivational Interviewing’ techniques, which approach the behaviour change process with empathy, a focus on understanding a patient’s motivations for change, and a goal of empowerment [11]. A brief intervention for reducing alcohol consumption would involve an assessment of drinking patterns that is then used to inform tailored advice and feedback on the behavioural and physiological effects of alcohol, risk of harm, and financial costs of alcohol consumption [12]. Brief interventions have traditionally been conducted in individual sessions in clinical settings such as hospitals [13], primary health care [14] and within substance disorder treatment contexts [15]. More recently, brief interventions involving face-to-face contact have been shown to reduce alcohol consumption in college and university students [16–18]; however, this mode of delivery is resource intensive and has limited reach. Alternative delivery methods are therefore needed to apply brief interventions to broader populations in the community.

Mobile phones offer a new method for reaching populations with health interventions. In Australia, 89 % of the adult population owns a smartphone, using them regularly for SMS and internet access [19]. These phones are a viable option for intervention delivery, with previous researchers reporting success in positively influencing sexual health, tobacco cessation, physical activity and healthy eating [20, 21]. Much of the available literature has utilised simple, one-way, untailored message dissemination, while brief interventions to reduce alcohol consumption require an assessment of current drinking behaviours. Therefore a suitable method of data collection is required if brief interventions are to be applied on a larger scale via mobile phone during drinking events.

Ecological Momentary Assessment (EMA) involves repeated observation of self-reported behaviour, in a participant’s natural environment, and permits collection of data during alcohol consumption events [22–24]. Real-time assessments of drinking require low cognitive demand and reduce the recall bias seen in retrospective reporting of alcohol consumption [24]. Several researchers have successfully implemented EMA using a mobile phone platform. Kuntsche and Labhart [23] used EMA in a study-specific smartphone application to record the alcohol consumption of young Swiss people, with SMS reminders sent throughout the drinking event. Riordan, Scarf and Conner [25] used SMS to collect alcohol consumption data nightly throughout orientation week for university students in New Zealand. Suffoletto et al. used SMS to collect weekly drinking intention (prior to drinking) and recalled drinking data from young people, with tailored feedback sent in response [26–28]. However, we could find no studies that have examined whether EMA during a drinking event could underpin the delivery of an immediate brief intervention.

The combination of EMA and brief interventions has further potential benefits in the timing of intervention delivery. Brief interventions are generally undertaken outside of drinking events, targeting overall alcohol consumption. However, drinking is a contextually bound behaviour [24] and few studies have attempted to intervene during risk events, where the personalised feedback that characterises many brief interventions could be relevant and timely. While this combination shows promise in concept, it is not known whether it is feasible and acceptable to combine data collection and brief interventions during drinking events.

### Aim of study

To investigate the feasibility and acceptability of mobile phone-delivered data collection and intervention for young people during drinking events.

## Methods

The study was approved by the Monash University Human Research Ethics Committee. The consolidated criteria for reporting qualitative research [29] guided the research to improve rigour and transparency in reporting.

### Study design

We employed a mixed-methods participatory design involving three stages of data collection. Firstly, focus group-style development workshops were held to explore an initial intervention design and inform the creation of intervention content. The proposed intervention was then redesigned and refined on the basis of these workshops. Secondly, these same participants tested the intervention during a regular night out on which they planned to drink. Finally, we evaluated the intervention using a mobile survey and in-depth interviews to canvass participants’ opinions of the intervention. In this paper we focus on design factors related to feasibility and acceptability. Data pertaining to the development of message content within this study will be the subject of a future publication.

The research was conducted in metropolitan Melbourne, Australia, with all interviews and focus groups/workshops occurring at the authors’ institution. Recruitment and workshops were completed in June 2014. Pilot testing of the intervention occurred between November and December 2014, with follow-ups occurring approximately one week after testing.

The research team was comprised of qualified experts in health promotion, interventions using new technology, and alcohol consumption, including specific expertise in the young adult population group. All team members were involved in the development and refinement of data collection instruments throughout the study. The researcher responsible for conducting interviews and focus groups has training and experience in qualitative methodology. A senior team member with extensive experience in qualitative research and participatory methods reviewed transcripts to verify findings in the analysis stage.

### Study population and recruitment

Participants were aged between 18 and 25 years, owners of smartphones, and self-reported ‘regular’ consumers of alcohol (drinking at least once per week on average). No further inclusion/exclusion criteria applied. Two methods of recruitment were utilised to generate a sample: firstly, 64 young people who had completed a questionnaire at a music festival [30] and indicated an interest in participating in other studies were sent a text message with some brief details and an invitation to contact the primary researcher for more information; 11 participants were recruited through this method. A further 37 participants were recruited through advertising placed at universities and through other community organisations working primarily with young people, as well as on social media. Six participants interested in participating withdrew prior to the research commencing, primarily due to being unavailable at the four sessions scheduled. The final sample of 42 young people, all of whom attended the development workshops, included 21 men and 21 women. Contact with participants outside of the workshops/interviews was exclusively electronic, with the majority occurring via text message, in addition to an electronic poll used to indicate availabilities for the workshop, intervention testing, and follow-up. Participants could also contact the researcher via phone call or email, but none did so. Participants received a cash reimbursement of AUD$150 for their participation; this was in compensation for the use of their phone data in the trial as well as their time. Reimbursement was not dependent on completing SMS assessments, and participants were free to withdraw at any point. Written informed consent was obtained from all participants.

Of the 42 young people, 40 were retained throughout all stages of the study, with two participants attending a workshop but not completing the intervention testing or follow-up. One was lost to follow-up and another moved overseas, resulting in a retention rate of 95 % throughout the three stages of the study.

Participants were predominantly Caucasian (82.5 %). Most (81 %) participants were students, of whom 79 % were undergraduate university students, with the remainder postgraduates (18 %) or vocational/technical college students (3 %). In terms of highest level of completed education, around two-thirds (63 %) had completed high school, 2 % had completed a vocational/technical course and the remainder (34 %) had completed an undergraduate degree.

### Data collection methods

#### Development workshops

Participants were split into four workshop sessions scheduled according to their availability. Between seven and 12 participants attended sessions, each facilitated by one researcher. Each session ran for approximately three hours and was structured to include a focus group-style discussion of the proposed research design; a media analysis component in which participants discussed and evaluated various styles of alcohol messages used in previous anti-alcohol campaigns and interventions; and a design session in which participants were broken into groups of three or four and given printed materials to help them design their optimal versions of the research and message content.

Participants were informed that the study was designed to design and test an intervention to reduce alcohol consumption in young people through the repeated collection of alcohol consumption and contextual information (with the example given of location) via mobile phone during their night out, that would be followed by tailored SMS feedback in response to each round of data collection. Participants were asked to express ideas and opinions on acceptability, feasibility, preferred data collection methods (e.g. sending data directly via text, SMS with web-survey link, or smartphone application), question content and wording, foreseeable barriers, optimal timing and frequency of data collection, and alcohol-related health messaging. We asked participants to generate ideas for question content to inform the research team as to how to best tailor the feedback to reduce alcohol consumption for themselves and others of their age. In addition to focus group-style discussion, each participant was given the opportunity to write down opinions on optimal timing, frequency, platform and content of the intervention. All sessions (including the design sessions in smaller groups) were recorded digitally and transcribed verbatim; thematic analysis of transcripts and documents (see below) began after the first workshop and was used to assist with probing questions in subsequent sessions.

#### Intervention refinement and content development

Following the completion of the workshops and thematic analysis, the full research team decided how to implement design changes and develop message content using theoretical frameworks. The process of redesigning the data collection and intervention included negotiation of practical and logistical considerations and the incorporation of new ideas generated through workshops to ensure feasibility and acceptability. The message content was developed into a matrix of messages classified according to appropriateness for location, gender, stage of night and variables collected from the EMA; classifications were informed by the workshops.

#### Intervention testing

Testing occurred approximately four months following the development workshops, on nights selected by the participants. Informed by stage one, the data collection involved a mix of text-message and mobile-compatible web questionnaires. The intervention involved participants nominating one single night within a two week period, on which they had social plans and were likely to consume alcohol. When scheduling their test night, participants pre-nominated what time they wished the surveys to begin, with most opting to complete the pre-survey at 7 pm. From their nominated start time, they were sent a link via SMS to the first mobile questionnaire which collected contextual data on plans for the evening, goal-setting (number of drinks and spending), if they had eaten, mood, motivations for drinking less (e.g. health/fitness, avoiding hangover, spending too much, not waking up in time for planned activities etc.), if any alcohol had been consumed so far, and with the option of writing a message to themselves to be sent later in the evening. Hourly SMS were then sent with links to a shorter EMA, which collected data on alcohol consumed since last data was sent, spending, location, how intoxicated (if at all) they perceived themselves to be and current mood. Each questionnaire allowed participants to opt out for the evening. The following day, at 12 pm, participants were sent a follow-up questionnaire that collected any missed data from 2 am onwards, and asked participants to try to recall total number of standard drinks consumed, total spend, and any adverse events due to drinking.

**Fig. 1:**
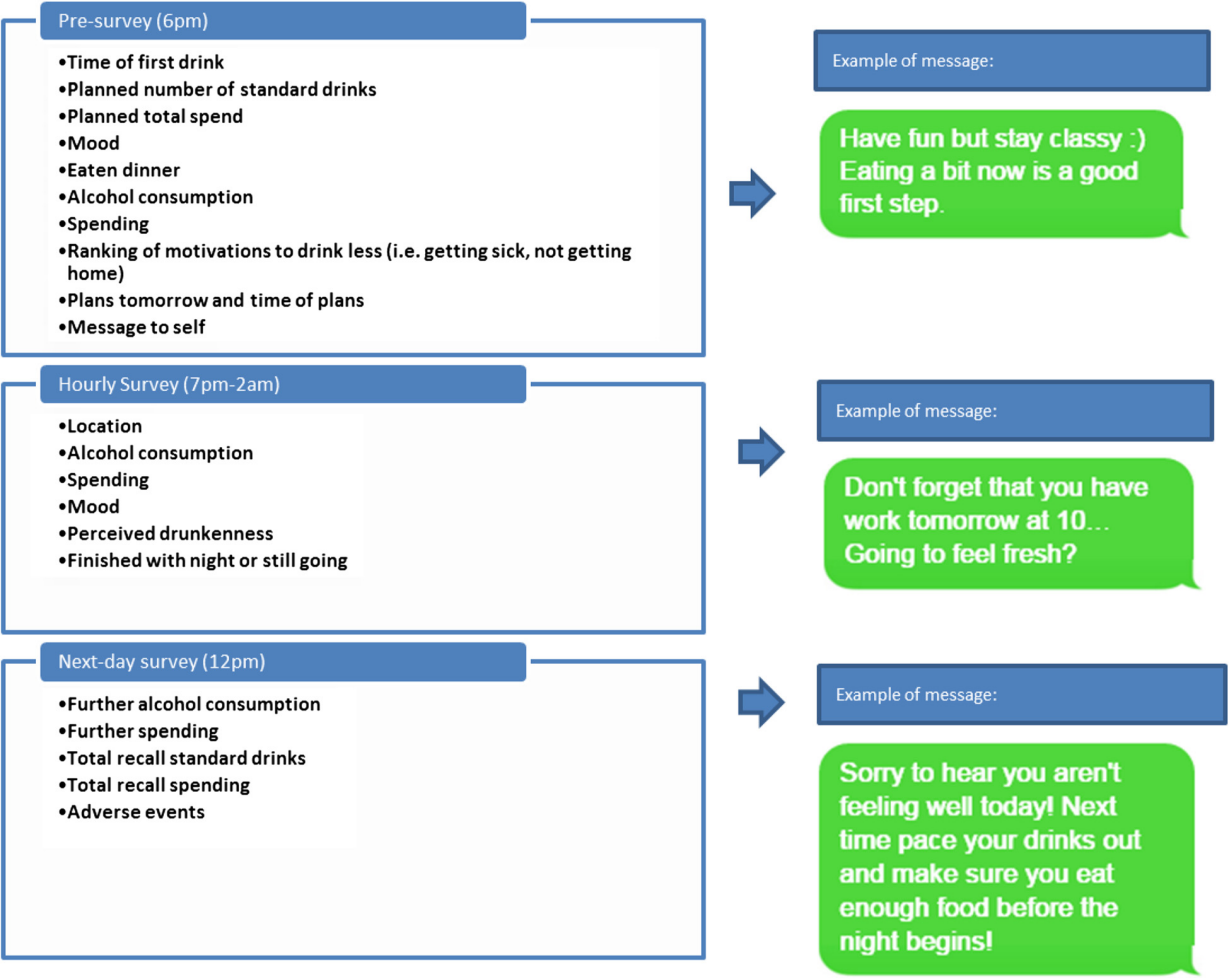
Variables collected throughout drinking event with message examples

Each time a participant responded to a questionnaire, they received a manually-tailored SMS message according to one or more of the following: gender, goals and plans set, amount of alcohol consumed so far, amount spent, location, priorities (as determined by what they reported might motivate them to drink less) or a message that they had written to themselves. Using the pre-developed matrix, the researcher identified an appropriate feedback message based on the participants’ reporting, and sent the message using online SMS tool Qmani (www.qmani.com). While this manual process has obvious limitations for scalability, the researchers felt it allowed them to better investigate the tailoring process, and will use the findings to build an automated system in future research. Participants were not aware that the messages were tailored manually. A feedback SMS was intended to be sent immediately after completion of every EMA, however, in practice, the manually tailored response took 1–2 min. For example, a participant completed a survey stating that they were still out at a nightclub/bar, had ranked ‘not getting home’ as high on their priorities of events to avoid, and had exceeded the number of drinks that they planned to have. Based on these responses, the researcher used filters to identify the following message in a matrix cell: “You’ve already had more than you planned to drink tonight. Have you got a lift home planned?”. Figure 1 illustrates the variables collected in each survey with further examples of messages.

No participant received the same message twice. The feedback for the next-day survey included tips for their next night out, or summary of total spend or alcohol consumed compared to goals or the recall reported. Self-reported alcohol consumption data collected during the event were not analysed in depth due to the small sample. Data pertinent to feasibility and acceptability were collected in the form of response rates, complemented by rich qualitative data collected in the evaluation stage. As mentioned, the development and evaluation of the message content will be described in a future publication.

#### Evaluation

Participants were followed-up approximately one week after completing their trial of the intervention. They were asked to attend a one-on-one interview or small focus group; either took approximately an hour. Each participant completed a questionnaire on their mobile phone with question items capturing demographic data, preferred time points and frequency, the invasiveness of the trial, and 5-point Likert rating scales (1 = very poor, 5 = very good) for user friendliness, questionnaire design, visual appeal, ease of use, phone compatibility, questionnaire length and question clarity. The interviewer then used a semi-structured approach to gain further feedback and allow the participant to elaborate on questionnaire responses, propose new ideas and discuss their experience of trialling the intervention. Finally, participants were asked to evaluate the tailored messages received during the pilot testing, including message suitability, usefulness and language. Experiences of responses to different messages were then discussed in depth, and the opportunity to modify content was offered. Member checking was completed in interviews and focus group to ensure that researchers’ interpretations matched the intended meaning of the participants’ feedback. Field notes were also taken during these sessions. We used these qualitative and quantitative data to investigate feasibility, acceptability, optimal timing and frequency of data collection and feedback, scalability and experiences for the trial.

### Data analysis

Transcripts, design material produced by participants and field notes were analysed thematically in an iterative process of coding, using NVivo V10 (QSR International, Melbourne, Australia). Due to the intended practical application of the findings, specific codes were generated in advance (such as that relating to optimal timing), while others emerged from the data during the data collection and analysis process. A second researcher cross-coded a sample of transcripts to verify the analysis framework.

While the aim of the current study was not to test the effectiveness of the intervention, we analysed descriptive process data from the testing phase, including response rates, in SPSS Version 22 (IBM, Armonk, USA). We also examined quantitative data from the evaluation survey on design features such as timing and frequency, complementing in-depth qualitative evaluation of the intervention and research; this is known in mixed-methods research as data triangulation and improves the rigour of research by providing multiple data sources [31].

## Results

Results are grouped below according to three domains: acceptability factors, feasibility considerations and participants’ experiences of the intervention.

### Acceptability of the intervention

#### Development workshop

The young people described feeling comfortable with reporting on alcohol consumption throughout drinking events. Participants also reported that it would be acceptable to regularly report on location, and occurrence of drinking-related adverse events, including vomiting, violence, accident/injury and sex, among others. In each of the four groups, at least one participant suggested adding adverse events to the list of options such as illegal behaviours such as drug-taking and drink-driving. When questioned about privacy with respect to these sensitive behaviours, most were unconcerned, with one male replying *“I guess you just, like, know that you’re not asking to rat us out. So you’d just say it”*.

Participants were asked to suggest other acceptable and important question items, either to help us to understand the nature of their nights out or to allow us to provide tailored feedback intended to reduce harmful drinking. Questions on spending, mood, plans and priorities were added as a result, but in the newly developed format of a separate and longer first questionnaire, a regular EMA to be completed hourly with fewer questions, and a post-intervention questionnaire to be completed the following day. Most participants they did not mind answering slightly longer questionnaires at the beginning of the night or the following day, as long as the hourly EMAs were brief enough to not detract from their social enjoyment.

Participants agreed that mobile phones were very suitable for both data collection and message dissemination, as young people are rarely far from their phones. Many participants stated *“I’m on my phone anyway”.* However, as indicated above, acceptability hinged on design factors that determined convenience.

#### Testing and evaluation

Following the night of trialling the intervention, most participants retained their views relating to its acceptability. Many participants noted the non-judgmental approach of the broad project, which they believed encouraged participation. Many recognised that this type of research had the potential to feel burdensome if not designed and framed carefully. One participant stated in the follow-up interview that he had been sceptical that the SMS feedback might “*feel like someone was nagging me when I just want to have fun”,* but instead found the experience more positive: *“I felt like someone was just checking up on me. It was sort of nice (laugh).”* The casual language used in question wording was also identified as important. Wording in the evaluation questionnaire was regarded positively, rated a mean of 4 out of 5, with participants describing the language as appropriate, clear and relatable. Of the 40 participants, 31 opted to enter in a “message to self” to be sent back to them at a later time. This option was described by participants positively in the evaluation, as it allowed them to enter in an entirely personal note or motivation.

Almost all (98 %, *n* = 39) evaluation survey participants indicated that they were comfortable responding to all questions included in the pilot, which was confirmed in follow-up interviews. Furthermore, the research was described as being socially acceptable to friends and others around them on the night, with only 5 % (*n* = 2) stating that they wouldn’t want their peers to know that they were tracking and reporting their drinking. When questioned further on this, some participants indicated that they had told friends as they saw it as novel, whereas others had warned friends in advance that they might be slightly more distracted than usual. In the evaluation survey, participants were asked to measure invasiveness in terms of whether they felt that completing the trial interrupted their night, with 75 % (*n* = 30) disagreeing or strong disagreeing on a four-point Likert scale with the statement “Doing the trial interrupted my night too much”. Further, when asked if doing the trial interrupted their night “a little”, “a lot” or “not at all”, only 2 % selected the option “a lot”, with 83 % selecting “a little” and 15 % selecting “not at all”.

In evaluation interviews, participants mostly reported that during testing, they were interested to see what the feedback message would be generated based on their submission, although some suggested that this would be improved if available within seconds following data entry. Feedback SMS were generally sent within two minutes during the trial but any reduced interruption was seen as beneficial. In addition, almost all participants reported that they re-read the feedback SMS the following day, while three-quarters reported sharing messages with one or more friends when they received it.

### Co-designing feasible research

#### Development workshop

Designing minimally-invasive data collection tools was pivotal to the logistical feasibility of repeatedly collecting data through a drinking event. Creating a purpose-specific smartphone application for the intervention appealed to approximately half of the young adults, but many participants expressed concerns over compatibility between phones and mentioned that they would probably ignore an application notification, so we chose to use SMS and links to web-based surveys. Almost all participants agreed that they were more likely to read an SMS with urgency than other contact methods, with one explaining *“You don’t really get spammed by text. So it’s probably a friend and so you kind of feel like you have to read it and reply. I reckon I’d just do it straight away because that’s how you think”.* However, participants cautioned against submitting data via text or having to type out responses as it was more labour intensive, prone to errors and especially difficult while drinking. Compatibility, visual appeal and ease of response were still seen as barriers to completing questionnaires in web browsers. As a result, we tested several online survey tools before settling on SurveyGizmo due to its mobile compatibility. Several iterations of the questionnaire were pre-tested on various models of smartphones by over 20 researchers and 12 young adults who did not participate in the main study.

Determining the most appropriate frequency and timing of questionnaires required participants to consider invasiveness against what was most likely to capture alcohol intake through the evening. Hourly surveys were seen as preferable; most participants agreed that spacing questionnaires further apart than one hour would result in difficulties recalling drinks and spending, while more frequent questionnaires were expected to be too invasive. Other suggestions were also made, including allowing participants to determine the frequency in the pre-survey, based on their own pace, and the option of participants sending back information each time they bought a drink. The consensus across groups was that the first questionnaire should be sent at 6 pm and the last at 2 am, with the option to set a later start time and to request an earlier opt-out. This timeframe was expected to cover the majority of drinking events.

The feasibility concern most frequently discussed in the workshops related to the measurement of alcohol consumption. Most participants reported lacking confidence in calculating or reporting units of standard drinks, and many agreed that they would not be able to recall number of standard drinks consumed on a typical night, presenting a clear challenge to the design of the research.

#### Testing and evaluation

Technical difficulties during the pilot were few and minor, with all SMS successfully delivered, and only one glitch (related to SurveyGizmo updating their system) that prevented some participants from moving to the second page of the first questionnaire; this was resolved reasonably quickly. We sent 295 SMS prompts, resulting in 262 completed questionnaires (89 %). In evaluation interviews, explanations for missed rounds of data collection included phones being on silent, finishing work later than expected, phone running out of battery, forgetting and being in an inappropriate social situation, and the technical glitch. Table 1 describes the response rates across the hourly intervals.

**Table 1:** Response rates per hour in intervention test

Time sent	Number of surveys sent	Number of surveys completed	%
Pre-survey	40	40	(100 %)
8 pm	38	34	(89 %)
9 pm	37	30	(81 %)
10 pm	38	31	(82 %)
11 pm	35	32	(91 %)
12 am	29	22	(91 %)
1 am	22	20	(91 %)
2 am	12	10	(83 %)
12 pm (next day)	40	40	(100 %)
Total	295	262	89 %

In terms of surveys completed per individual, 21 of the 40 participants completed all surveys sent to them, 10 missed only one survey, five missed two surveys, and four missed more than two surveys.

Questionnaire design was rated highly, with 90 % (*n* = 36) of survey respondents agreeing that completing them was easy. Qualitative evaluation indicated that the questionnaire displayed well across all but one phone type (a very old smartphone model).

Despite most participants opting to start surveys from 7 pm on their testing night, the evaluation showed a preference for 6 pm commencement so to complete goal-setting prior to any alcohol consumption, and before they may be out for dinner.

Following testing, most participants (68 %) still advocated for hourly questionnaire frequency; remaining participants suggested half-hourly (13 %), every hour and a half (7 %) or every two hours (12 %). In interviews, participants more strongly recommended the option of user-determined frequency, or diary-style data entry. Both survey and interview data supported the timing chosen for data collection.

As informed by development workshops, alcohol consumption was measured through a series of questions asking what types of drinks were consumed (e.g. beer, cider, wine, spirits), and then quantity in different units of each (e.g. pot, pint, bottle, longneck, shot). These responses were converted to standard drinks based on average alcohol concentration in different drink types. While participants explained in interviews that this was simple enough in terms of data entry, concerns were expressed relating to the accuracy of data. Some apprehension was based on difficulties in recalling what they had consumed in the last hour (e.g. *“I couldn’t remember if I’d already reported it in the last round or not”*), while other concerns related to consuming higher-strength drinks, or using non-standard glass sizes. When asked to recall total standard drinks consumed during the night, participants reported an average of three fewer drinks (mean = 3.16) than had been recorded throughout the drinking event in EMAs.

Tracking of spending also proved challenging, with 61 % of survey participants agreeing that calculating hourly spending was difficult. Interview participants explained that they experienced most difficulty when drinking in a home-based setting, drinking pre-purchased alcohol, or when ‘shouting’ rounds of drinks for others.

### Expectations of effectiveness for reducing drinking

#### Development workshop

Focus group discussion indicated that most participants had recent and regular experiences of drinking heavily and, initially, none reported being interested in reducing alcohol consumption. However, despite the acceptance of binge drinking as a regular practice, there was notable curiosity and interest in attempting to track drinking as many admitted that they took little notice of their consumption in most drinking events. Several participants across groups reflected similar sentiments, stating that they *“probably don’t know where to stop”* and that *“… It can get out of hand sometimes”*, and telling the common story of the night going well until the final part of the evening, when poor decision-making occurred*.* For our young participants, motivations for reducing alcohol consumption centred on minimising this poor decision-making rather than any concerns for health or safety.

One participant wished she *“had a sober version of myself, keeping check”*, while others recalled needing a sober friend to assist them in making responsible decisions. In this sense, tracking alcohol consumption and receiving positive and practical tailored messages were seen to be potentially very useful on ‘bigger nights’. A small number of male participants in one group were apprehensive about how they might react, and discussed the risk of responding to messages defiantly by drinking more. However this was agreed to be more likely if messages were written in a didactic tone and explicitly instructed recipients not to drink.

The participants were very interested in reducing spending on alcohol; without prompting, members of each group hypothesised that an intervention focused on tracking spending would be as effective, if not more effective, than standalone alcohol tracking. One participant stated that messages should: *“Hit me where it hurts, in the hip pocket”*, with a large majority of others agreeing that this approach had good potential to reduce drinking.

#### Testing and evaluation

While this pilot study was not designed to test the intervention’s efficacy in reducing alcohol consumption, feedback from evaluation interviews showed moderate to strong support for the intervention. When asked to recount experiences of trialling the intervention, several encouraging themes emerged.

Firstly, recording their own alcohol consumption necessitated an attention to drinking that most participants had never previously attempted. In the evaluation survey, 84 % of participants agreed or strongly agreed on a four-point Likert scale that completing the intervention “helped me keep track of my drinking and spending”. This was described by one young woman as *“a bit of an eye-opener”*, while several others reflected that on subsequent drinking occasions they had been noticing their intake more closely. Setting a goal at the start of the night for maximum number of drinks was also something new to many participants, which some reported as useful.

Secondly, while spending was regarded as difficult to track, it was still seen as a motivation for reducing drinking. Setting goals for spending was similarly regarded as new and potentially useful, while some participants reported receiving reminders when they had gone over this limit encouraged slowing or stopping drinking altogether: *“I got the message saying I’d spent all my money, and then I don’t know what happened but I was just like ‘I’m done’”.* Likewise, reminders informing participants that they had reported having plans the next day was described as a potentially important tool with some promising anecdotal effects: *“I totally forgot I had to work the next day and the message said I had to start in six hours so that was good.”*

## Discussion

Our study demonstrated that young adults are both willing and able to engage in mobile-delivered research and interventions targeted to them during drinking events. Although further refinement is required to enhance the validity of data collected through a drinking event, our sample of young people assessed the process of collecting these data and providing relevant feedback as useful for reducing drinking and associated harms.

### Acceptability

Young people described themselves as comfortable to report drinking data and were unconcerned about privacy, even when reporting more personal information including goals and priorities, location, spending and the occurrence of adverse events and behaviours such as drink-driving or drug use. While the willingness to report on such a wide variety of factors using mobile phones was surprising, it doubtless reflects the amount of time young people typically spend on their phones and the comfort and familiarity that young adults have with sharing personal information over technology. This is encouraging for future studies intending to intervene during risky drinking events. The wide range of questions suggested for inclusion in the EMA reflected the complexity of participants’ drinking events, shaped by social context and varying motivations and priorities for reducing drinking. Participants recognised the need for the researcher to have better insight into their context, and be able to ‘speak their language’, to make an intervention truly relevant. The combination of data collection and intervention during drinking events therefore presents a promising avenue of intervention that requires further testing and evaluation.

Participants initially anticipated that our data collection and intervention could intrude too much during social events to be successfully implemented. However, we demonstrated that if data collection procedures are co-designed by young people and tested intensively, intrusion can be minimised to an acceptable level. Crucial to this was a design that allowed easy and rapid data collection and for feedback messages to be sent almost immediately. Most participants valued receiving a feedback message after data collection, seeing it as little added burden.

### Feasibility

We initially expected challenges relating to phones running out of battery, poor reception, participant non-response, and technological errors such as SMS not being received or the questionnaire not displaying correctly. However, in line with previous studies, our response rate of 88 % suggests that it is feasible to collect data during drinking events. Irvine et al.’s [32] intervention for reducing alcohol consumption in disadvantaged men had a response rate of 88 % to question-based text messages. In a six-month study with weekly reporting of alcohol consumption, 82.1 % of participants completed all EMAs [33]. In another study requiring daily completion of EMAs, Kuntsche and Labhart [23] reported an 80.4 % completion rate. This suggests that the addition of brief intervention to EMA did not impact on participation in the study, although further research is required to confirm this. It is expected that collecting data over multiple nights will result in higher attrition, although participants reported in follow-up interviews that they were willing to participate in repeated nights of data collection if requested, as long as they were able to choose the nights involved.

SMS was regarded as the best notification system due to its perceived urgency, and few problems were experienced in using a web-based mobile survey. Some participants suggested the intervention be moved to a smartphone application platform, and this is worth exploring in future research. A recent review of drinking-related smartphone applications showed that some have similar functions of tracking alcohol consumption and providing feedback [34]. However, most current apps appear designed to encourage increased alcohol consumption rather than promoting harm reduction. Our combination of EMA and brief intervention would provide an alternative to these if further developed for an app platform.

In order to best capture alcohol consumption over the night without intruding too heavily, hourly EMAs between 7 pm-2 am were preferred. The greatest challenge to feasibly conducting the research lay in the reporting of alcohol consumption due to non-standard units of alcohol and difficulty in recall; this is not an issue that has been discussed in previous research using EMAs. It is not known if the mean difference of three drinks between standard drinks reported the following day and drinks reported during the night was due to inaccurate reporting during the night or loss of memory. However, previous research has shown that EMAs can reduce recall bias and improve reliability and validity. Other research has shown similar discrepancies, with higher reporting of alcohol consumption in EMAs and lower retrospective recall [24]. Our and Monk et al.’s [24] studies also reported qualitative data indicating that many participants relied on guessing to report retrospective recall of alcohol consumption, due to memory impairments or confusion. We also found reporting of spending was not straightforward, with particular challenges related to pre-purchased or shared drinks. Nevertheless, despite potential inaccuracies, participants still reported value in the tracking process. Future research needs to determine and test the best ways to capture data relevant to alcohol events.

### Expectations for the intervention to reduce drinking

Participants who did not report a desire to reduce alcohol consumption still expressed a desire for assistance in retaining control over drinking and decision-making in the later part of drinking events. This finding highlights potential areas for intervention targeting, although messages must be designed in a way that engages participants. While qualitative reports from the pilot intervention are not evidence of effectiveness, participants did describe experiences that suggest different pathways for intervention; these include raising awareness of an individual’s own consumption and spending, or by reminding people of their sober self, as well as providing decision-making support based on their pre-reported personal motivations and priorities. These pathways provide multiple mechanisms through which the combination of EMA with brief intervention could influence RSOD behaviour, and further exploration of these is warranted.

## Limitations

This study is constrained by its relatively small purposive sample, meaning that results are not necessarily generalisable to a broader population. Further, all data are self-reported and thus subject to responder bias; social desirability bias and dominant responder bias are particularly pertinent to the development workshops, although measures were taken in facilitation to ensure that participants had equal opportunity to contribute. Moreover, data collected while participants were under the influence of alcohol may be prone to additional recall bias; however, these data were collected for the purposes of producing tailored feedback as opposed to generalising results. Finally, the high level of engagement shown by the young people involved was also likely to have had a positive influence on response rates, and it is not known if this would be replicated in other study populations, for instance less well-educated young people. Further, the intervention was only tested on one night, and a higher rate of attrition may occur over multiple nights of testing.

The study has several strengths, including its participatory design to inform and refine intervention design. Further, the study adds to the evidence base by providing transparent detail regarding the rigorous development and design process, a gap noted in recent reviews of text message-based behaviour change interventions [35]. The mixed-methods design of the study allowed for a comprehensive intervention development process. Rigour was aided by use of data triangulation, member-checking and cross-coding by researchers.

## Conclusion

The study illustrates the use of a participatory design for developing an intervention for reducing alcohol consumption for young people. Recommendations from participants led to the inclusion of broader contextual information within the questionnaires delivered through EMA, which improved the personalised feel of the intervention. The young people informed the frequency and timing of EMAs, as well as question content and language and other design features. Data from follow-up interviews and questionnaires will be used to further refine the intervention for future research. The intervention was largely perceived to be acceptable, feasible to upscale, with ease of use minimising invasiveness and underpinning high response rates. The promising experiences described qualitatively suggest that the combination of EMA and brief intervention may have the potential to positively influence drinking events. The study provides detail on the development process of an intervention delivered on mobile platforms, which the literature lacks. Further work is needed to test the efficacy of this type of intervention in reducing harms related to alcohol consumption events.
